# Recent Increase in COVID-19 Cases Reported Among Adults Aged 18–22 Years — United States, May 31–September 5, 2020

**DOI:** 10.15585/mmwr.mm6939e4

**Published:** 2020-10-02

**Authors:** Phillip P. Salvatore, Erisa Sula, Jayme P. Coyle, PhD, Elise Caruso, Amanda R. Smith, Rebecca S. Levine, PhD, Brittney N. Baack, Roger Mir, Edward R. Lockhart, Tejpratap S.P. Tiwari,, Deborah L. Dee, Tegan K. Boehmer, Brendan R. Jackson, Achuyt Bhattarai

**Affiliations:** ^1^Epidemic Intelligence Service, CDC; ^2^CDC COVID-19 Response Team; ^3^Oak Ridge Institute for Science and Engineering, Oak, Ridge, Tennessee.

Although children and young adults are reportedly at lower risk for severe disease and death from infection with SARS-CoV-2, the virus that causes coronavirus disease 2019 (COVID-19), than are persons in other age groups (*1*), younger persons can experience infection and subsequently transmit infection to those at higher risk for severe illness (*2*–*4*). Although at lower risk for severe disease, some young adults experience serious illness, and asymptomatic or mild cases can result in sequelae such as myocardial inflammation (*5*). In the United States, approximately 45% of persons aged 18–22 years were enrolled in colleges and universities in 2019 (*6*). As these institutions reopen, opportunities for infection increase; therefore, mitigation efforts and monitoring reports of COVID-19 cases among young adults are important. During August 2–September 5, weekly incidence of COVID-19 among persons aged 18–22 years rose by 55.1% nationally; across U.S. Census regions,* increases were greatest in the Northeast, where incidence increased 144.0%, and Midwest, where incidence increased 123.4%. During the same period, changes in testing volume for SARS-CoV-2 in this age group ranged from a 6.2% decline in the West to a 170.6% increase in the Northeast. In addition, the proportion of cases in this age group among non-Hispanic White (White) persons increased from 33.8% to 77.3% during May 31–September 5. Mitigation and preventive measures targeted to young adults can likely reduce SARS-CoV-2 transmission among their contacts and communities. As colleges and universities resume operations, taking steps to prevent the spread of COVID-19 among young adults is critical (*7*).

CDC receives patient-level COVID-19 data from jurisdictional health departments through a standardized CDC COVID-19 case report form.^†^ Data on probable and confirmed cases from 50 states, the District of Columbia (DC), and four territories (Guam, the Northern Mariana Islands, Puerto Rico, and the U.S. Virgin Islands) were analyzed to determine national trends among demographic groups during May 31–September 5, 2020.^§^ When available, date of symptom onset was used in calculations of weekly trends of case data; if symptom onset date was unavailable, an alternative date was used in the following descending order: specimen collection date, date reported to CDC, or episode date (California only).^¶^ Trends were analyzed nationally and by U.S. Census region.

Measures of weekly SARS-CoV-2 real-time reverse transcription–polymerase chain reaction (RT-PCR) testing volumes by age were obtained from COVID-19 electronic laboratory reporting data submitted by state health departments (37 states) and from data submitted directly by public health, commercial, and reference laboratories (13 states and DC)** when age was unavailable in state-submitted data. Testing data from U.S. territories were not included. Total number of tests was calculated as the sum of negative and positive test results. Testing volume represents individual tests, not the number of persons tested. Date of specimen collection or test order date was used in calculations of weekly trends in testing volume.^††^

Data on COVID-19 cases and RT-PCR tests were aggregated by calendar week. Subgroup analyses of case reports and tests were analyzed using two measures: 1) number of reported cases (or tests) per 100,000 population per week (termed incidence for cases), which accounts for differences in underlying population size but is affected by reporting lags and underreporting; and 2) percentage of all cases (or all tests) each week, which does not account for differences in population size but is less affected by reporting lags or underreporting (assuming that reported data do not differ in important ways from lagged data).^§§^ All analyses were conducted using R software (version 4.0.2; The R Foundation).

During August 2–September 5, 2020, a total 999,579 persons with COVID-19 with case report data were reported to CDC, 15.6% of whom were aged 18–22 years. National weekly COVID-19 incidence among persons aged 18–22 years increased 62.7% (95% confidence interval [CI] = 60.0%–65.3%) during the 4-week period August 2–August 29 from 110 to 180 cases per 100,000 before declining to 171 during August 30–September 5 (Figure 1). During August 2–September 5, weekly incidence increased most in the Northeast (144.0%; 95% CI = 131.5%–157.3%) from 53 to 130 per 100,000, and in the Midwest (123.4%; 95% CI = 116.1%–131.0%), from 111 to 247 (Supplementary Figure 1, https://stacks.cdc.gov/view/cdc/94198). Notably, in the Northeast, weekly incidence has remained below 53 cases per 100,000 in all other age groups since July 4. In the South, weekly incidence among persons aged 18–22 years increased 43.8% (95% CI = 40.0%–47.6%) from 115 to 166 cases per 100,000. Weekly increases were smallest in the West, where incidence declined initially until August 22 and then increased through September 5, but, overall, declined 1.7% during August. During August 2–September 5, the proportion of all cases per week that occurred among persons aged 18–22 years approximately doubled (2.1-fold; 95% CI = 2.1–2.2), from 10.5% to 22.5%.

**FIGURE 1:**
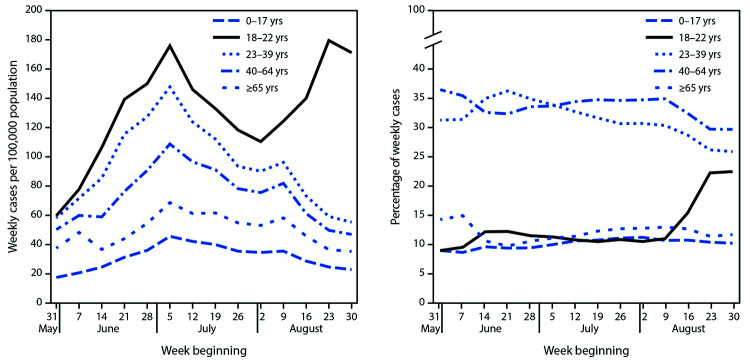
Weekly COVID-19 incidence in case surveillance data,* by age group — United States,^† ^May 31–September 5, 2020 **Abbreviation:** COVID-19 = coronavirus 2019. * From CDC COVID-19 case report surveillance systems. Case report surveillance systems record 76% of national aggregate case counts reported to CDC, based on an analysis of data reported during March15–August 15. ^†^ Includes cases in 50 states, District of Columbia, and four territories: Guam, the Northern Mariana Islands, Puerto Rico, and the U.S. Virgin Islands.

The number of weekly tests performed among persons aged 18–22 years increased 49.3% (95% CI = 48.7%–49.9%) from 1,877 tests per 100,000 during the week of August 2–August 8 to 2,802 during the week of August 30–September 5 (Figure 2). The largest increase in testing relative to population size was in the Northeast, where weekly tests increased 170.6% (95% CI = 168.3%–172.9%) from 1,975 per 100,000 to 5,345, and in the Midwest, where weekly tests increased 65.2% (95% CI = 63.9%–66.5%) from 2,264 per 100,000 to 3,740 (Supplementary Figure 2, https://stacks.cdc.gov/view/cdc/94197). In contrast, more modest increases were observed in the South (7.0% [95% CI = 6.3%–7.7%], from 2,041 to 2,183 per 100,000); and in the West, testing volume declined 6.2% (95% CI = 5.1%–7.2%), from 1,191 per 100,000 to 1,118. At the end of this period, the proportion of all tests performed nationally among persons aged 18–22 years had increased from 9.4% to 14.4% (1.5-fold [95% CI = 1.53–1.54] higher than at the beginning).

**FIGURE 2:**
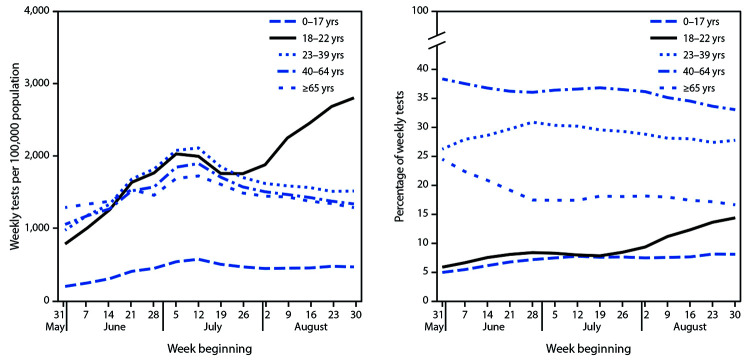
Total weekly SARS-CoV-2 reverse transcription–polymerase chain reaction (RT-PCR) test volume and percentage of weekly tests,* by age group — United States,^† ^May 31–September 5, 2020 **Abbreviation:** COVID-19 = coronavirus 2019. * Percentage of weekly tests was calculated as number of tests within each age group divided by number of tests in all age groups. Specimen collection date or test order date was used for analysis. Tests volume data were obtained from COVID-19 electronic laboratory reporting data submitted by state health departments for 37 states and, when age was not available in state-submitted data, from data submitted directly by public health, commercial, and reference laboratories for 13 states and the District of Columbia. The data might not include results from all testing sites within a jurisdiction (e.g., point-of-care test sites) and therefore reflect the majority of, but not all, SARS-CoV-2 RT-PCR tests in the United States. ^†^ Includes tests conducted in 50 states and District of Columbia.

When examined by race and ethnicity nationally, during August 2–September 5, the weekly incidence among White persons aged 18-22 years increased 149.7% (95% CI = 78.8%–248.7%), from 48 per 100,000 to 120 (Figure 3). During May 31–June 20, the proportion of weekly cases that occurred among White persons aged 18–22 years increased from 33.8%% to 50.8%. Then, during August 2–September 5, the proportion was 1.5-fold that during May 31–June 20 (95% CI = 0.2–12.9), having increased from 52.1% to 77.3%. At the same time, incidence among persons of other racial and ethnic minority groups remained stable or declined. The largest increases in incidence among White persons were in the Midwest (198.2%; from 65 to 195 per 100,000) and the Northeast (168.4%; from 14 to 37 per 100,000) (Supplementary Figure 3, https://stacks.cdc.gov/view/cdc/94196).

**FIGURE 3:**
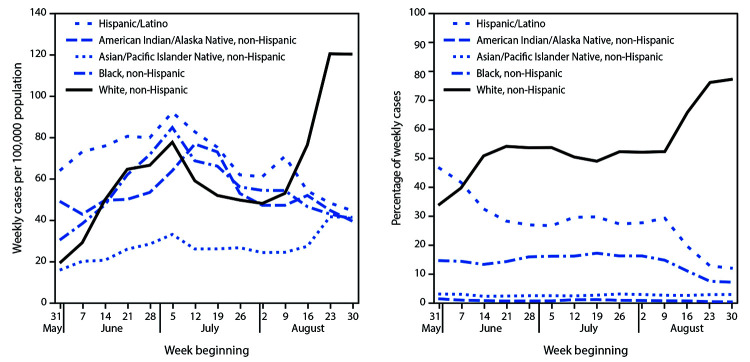
Weekly COVID-19 incidence in case surveillance data* among persons aged 18–22 years, by race/ethnicity^†^^,^^§^ — United States,^¶ ^May 31–September 5, 2020. **Abbreviation:** COVID-19 = coronavirus 2019. * From CDC COVID-19 case report surveillance systems. Case report surveillance systems record 76% of national aggregate case counts reported to CDC, based on an analysis of data reported during March 15–August 15. ^†^ Race/ethnicity data were not reported for 2,476,317 (48.5%) case reports; these cases were excluded from this subgroup analysis. ^§^ Race categories include persons of non-Hispanic ethnicity. ^¶^ Includes cases in 50 states, District of Columbia, and four territories: Guam, the Northern Mariana Islands, Puerto Rico, and the U.S. Virgin Islands.

## Discussion

In August 2020, CDC and case-reporting jurisdictions identified an increase in the percentage of COVID-19 cases among persons aged 18–22 years. Incidence in this age group changed 2.1-fold during this time, compared with a 1.5-fold change in testing (possibly related to new screening practices as colleges and universities reopened). Although increased incidence was likely driven in part by an increase in COVID-19 diagnostic testing, this is unlikely to be the sole reason for the observed increases in incidence. 

The observed increases in COVID-19 cases among persons aged 18–22 years could be driven by many factors, including changes in behavior or risk profiles resulting from multiple social, economic, and public policy changes during this period. Because approximately 45% of persons aged 18–22 years attend colleges and universities and 55% of those attending identified as White persons (*6*), it is likely that some of this increase is linked to resumption of in-person attendance at some colleges and universities. Detailed exposure information from patients in this age group (e.g., through targeted epidemiologic studies) can help identify the specific drivers of the observed trends.

The findings in this report are subject to at least four limitations. First, race/ethnicity data were complete for only one half of cases reported to CDC; changes in completeness of race/ethnicity data over time call for caution in interpretation of the observed trends in race/ethnicity. Second, data-reporting lags can delay recognition and reporting of trends in case surveillance data; for this reason, this report examines COVID-19 cases occurring through September 5, which might be more completely reported than are cases in more recent weeks. Third, a revised COVID-19 case definition introduced by the Council of State and Territorial Epidemiologists on August 5,^¶¶^ which updated definitions of probable cases, was gradually adopted by approximately one half of reporting jurisdictions during the period of this analysis and might have introduced additional variability in case reporting. Finally, trends in case surveillance data need to be interpreted in the context of laboratory testing patterns (e.g., repeat testing of all students in some university settings)*** and trends in other age groups and with evidence from other data sources; however, linking testing data with case surveillance remains a challenge because person-level data are deidentified before aggregation or analysis.

Previous reports identified young adults as being less likely than are other age groups to adhere to some COVID-19 prevention measures (*8*), which places them and their close contacts at higher risk for COVID-19. Approximately 71% of persons aged 18–22 years reside with a parent, nearly one half attend colleges and universities, and 33% live with a parent while enrolled (*6*,*9*). To prevent cases on campuses and broader spread within communities, it is critically important for students, faculty, and staff members at colleges and universities to remain vigilant and take steps to reduce the risk for SARS-CoV-2 transmission in these settings. Transmission by young adults is not limited to those who attend colleges and universities but can occur throughout communities where young adults live, work, or socialize and to other members of their households (*3*–*4*), some of whom might be at high risk for severe COVID-19–associated illness because of age or underlying medical conditions. Mitigation and preventive measures targeted to young adults (e.g., social media toolkits discussing the importance of mask wearing, social distancing, and hand hygiene) (*10*), including those attending colleges and universities, can likely reduce SARS-CoV-2 transmission among their contacts and communities. Institutions of higher education should support students and communities by taking action to promote healthy environments (*7*).

**Summary**

**What is already known about this topic?**

Young adults with COVID-19 can spread infection to their contacts and communities.

**What is added by this report?**

During August 2–September 5, 2020, weekly COVID-19 cases among persons aged 18–22 years increased 55% nationally. Increases were greatest in the Northeast (144%) and Midwest (123%). Increases in cases were not solely attributable to increased testing.

**What are the implications for public health practice?**

Young adults, including those enrolled in colleges and universities, should take precautions, including mask wearing, social distancing, and hand hygiene, and follow local, state, and federal guidance for minimizing the spread of COVID-19. Institutions of higher education should take action to promote healthy environments.

## Footnotes

* *Midwest:* Illinois, Indiana, Iowa, Kansas, Michigan, Minnesota, Missouri, Nebraska, North Dakota, Ohio, South Dakota, and Wisconsin; *Northeast:* Connecticut, Maine, Massachusetts, New Hampshire, New Jersey, New York, Pennsylvania, Rhode Island, and Vermont; *South*: Alabama, Arkansas, Delaware, District of Columbia, Florida, Georgia, Kentucky, Louisiana, Maryland, Mississippi, North Carolina, Oklahoma, South Carolina, Tennessee, Texas, Virginia, and West Virginia; *West*: Alaska, Arizona, California, Colorado, Hawaii, Idaho, Montana, Nevada, New Mexico, Oregon, Utah, Washington, and Wyoming. https://www.census.gov/geographies/reference-maps/2010/geo/2010-census-regions-and-divisions-of-the-united-states.html. Territories are not included in U.S. Census regions; therefore, cases from territories (<1% of national cases) were excluded from analysis of regional trends.

† COVID-19 case report form: https://www.cdc.gov/coronavirus/2019-ncov/php/reporting-pui.html.

§ Case report surveillance data record only 76% of national cases reported through aggregate count based on a daily, robust, multistep process to collect the data and confirm the numbers of cases and deaths within jurisdictions, ranging from 0%–100% in different states. Completeness calculations comparing case report surveillance data with aggregated nationally reported case counts were determined from cases reported during March 15–August 15 (extracted on September 4). In some cases, states report only confirmed cases in aggregate case counts to CDC, while providing case report data on probable and confirmed cases, resulting in >100% completeness. https://www.cdc.gov/coronavirus/2019-ncov/covid-data/faq-surveillance.html.

¶ The California Department of Public Health reports the date associated with COVID-19 as “episode date,” using the earliest date available from the following list: illness onset date, specimen collection date, date of death, or date reported. For all other states, date was classified using dates in the descending order of preference from the following list: symptom onset date; if illness onset date is not available, then specimen collection date; if specimen collection date is not available, then the date the case was reported to CDC.

** COVID-19 electronic laboratory reporting data submitted by state health departments from all laboratories performing SARS-CoV-2 RT-PCR testing were used for 37 states (Alabama, Alaska, Arizona, Arkansas, Colorado, Connecticut, Florida, Georgia, Hawaii, Idaho, Illinois, Indiana, Iowa, Kansas, Kentucky, Louisiana, Maryland, Massachusetts, Michigan, Minnesota, Montana, Nebraska, Nevada, New Hampshire, New Jersey, North Carolina, Oregon, Pennsylvania, South Carolina, South Dakota, Tennessee, Texas, Utah, Vermont, Virginia, West Virginia, and Wisconsin). SARS-CoV-2 RT-PCR testing data from a subset of public health, commercial, and reference laboratories were used for the District of Columbia and 13 states (California, Delaware, Maine, Mississippi, Missouri, North Dakota, New Mexico, New York, Ohio, Oklahoma, Rhode Island, Washington, and Wyoming). The data from the public health, commercial, and reference laboratories represent approximately 50% of all tests. The data might not include results from all testing sites within a jurisdiction (e.g., point-of-care test sites) and therefore reflect the majority of, but not all, SARS-CoV-2 RT-PCR tests in the United States. The data represent laboratory test totals, not individual persons, and exclude antibody and antigen tests.

†† Within COVID-19 electronic laboratory reporting data: state was assigned using location of the state health department that reported the test (available for 100% of tests), specimen collection date was used to assign date (available for approximately 98% of tests), and records with missing specimen collection data were excluded. Within data submitted directly by public health, commercial, and reference laboratories: state was assigned using patient location for 96% of tests, provider location was substituted for 1%, and records with both location fields missing (3%) were excluded; order date was used for 80% of tests, specimen collection date was substituted for 19%, and records with both date fields missing (1%) were excluded.

§§ Weekly incidence was calculated as the number of cases that occurred within a subgroup (age group or race/ethnicity) during each week, divided by the size of the U.S. population for that subgroup (in the same geographic region) using 2019 U.S. Census estimates, multiplied by 100,000. Similar methods were used to calculate weekly testing volumes per 100,000. Weekly percentage of total cases within a subgroup was calculated as the number of cases within that subgroup during each week divided by the total number of cases within all subgroups (and the same geographic region) during the same week. Similar methods were used to calculate weekly testing percentages (total positive and negative tests within a subgroup divided by total positive and negative tests across all subgroups and the same geographic region). For proportional increase in incidence (and proportional increase in testing volume), 95% CIs (reported as percentage increase for measures of number per 100,000 and as x-fold differences for measures of percentages) were calculated using the log-transformed delta method for ratios. Cases with missing values for reporting state/territory and symptom onset date (<1% and 1% of all case reports, respectively) were excluded. Race/ethnicity was categorized as “Hispanic/Latino” (all races) and separate non-Hispanic racial groups. Cases with missing values for age or race/ethnicity (<1% and 49% of all case reports, respectively) were excluded from analyses of those subgroups.
